# Global Mapping of Transcription Factor Binding Sites by Sequencing Chromatin Surrogates: a Perspective on Experimental Design, Data Analysis, and Open Problems

**DOI:** 10.1007/s12561-012-9066-5

**Published:** 2012-05-23

**Authors:** Yingying Wei, George Wu, Hongkai Ji

**Affiliations:** Department of Biostatistics, The Johns Hopkins Bloomberg School of Public Health, 615 North Wolfe Street, Baltimore, MD 21205 USA

**Keywords:** Transcription factor binding sites, DNase-seq, ChIP-seq, FAIRE-seq, Next-generation sequencing, Motif

## Abstract

Mapping genome-wide binding sites of all transcription factors (TFs) in all biological contexts is a critical step toward understanding gene regulation. The state-of-the-art technologies for mapping transcription factor binding sites (TFBSs) couple chromatin immunoprecipitation (ChIP) with high-throughput sequencing (ChIP-seq) or tiling array hybridization (ChIP-chip). These technologies have limitations: they are low-throughput with respect to surveying many TFs. Recent advances in genome-wide chromatin profiling, including development of technologies such as DNase-seq, FAIRE-seq and ChIP-seq for histone modifications, make it possible to predict in vivo TFBSs by analyzing chromatin features at computationally determined DNA motif sites. This promising new approach may allow researchers to monitor the genome-wide binding sites of many TFs simultaneously. In this article, we discuss various experimental design and data analysis issues that arise when applying this approach. Through a systematic analysis of the data from the Encyclopedia Of DNA Elements (ENCODE) project, we compare the predictive power of individual and combinations of chromatin marks using supervised and unsupervised learning methods, and evaluate the value of integrating information from public ChIP and gene expression data. We also highlight the challenges and opportunities for developing novel analytical methods, such as resolving the one-motif-multiple-TF ambiguity and distinguishing functional and non-functional TF binding targets from the predicted binding sites.

**Electronic Supplementary Material:**

The online version of this article (doi:10.1007/s12561-012-9066-5) contains supplementary material, which is available to authorized users.

## Introduction

One major goal of functional genomics is to comprehensively characterize the regulatory circuitry behind coordinated spatial and temporal gene activities. In order to achieve this goal, a critical step is to monitor downstream regulatory programs of all transcription factors (TFs). With the capability of mapping genome-wide transcription factor binding sites (TFBSs), chromatin immunoprecipitation coupled with high-throughput sequencing (ChIP-seq) [2, 21, 25, 29] or tiling array hybridization (ChIP-chip) [6, 27] have become standard approaches for studying gene regulation. Both technologies are now being widely used by investigators worldwide as well as consortium projects such as the ENCODE [9], modENCODE [7] and Roadmap Epigenomics [3] to map functional cis-regulatory elements. Although ChIPx (i.e., ChIP-seq and ChIP-chip) offers the power to survey genome-wide binding sites, a number of limitations make this technology low-throughput with respect to surveying a large number of TFs. First, successful application of ChIPx requires high-quality antibodies specifically recognizing the TF of interest. Unfortunately for many TFs, ChIP-quality antibodies are not available. Second, each individual ChIPx experiment can only analyze one TF in one cell type. To analyze many TFs, one has to test to ensure sensitive antibodies, optimize the protocol, and perform experiments repeatedly, which is both costly and labor-intensive. For these reasons, currently it is unrealistic to use ChIPx to directly monitor genome-wide TFBSs for all TFs. Therefore, the development of innovative methods and technologies that allow high-throughput mapping of in vivo TFBSs of all TFs is both important and urgently needed.

**Fig. 1:**
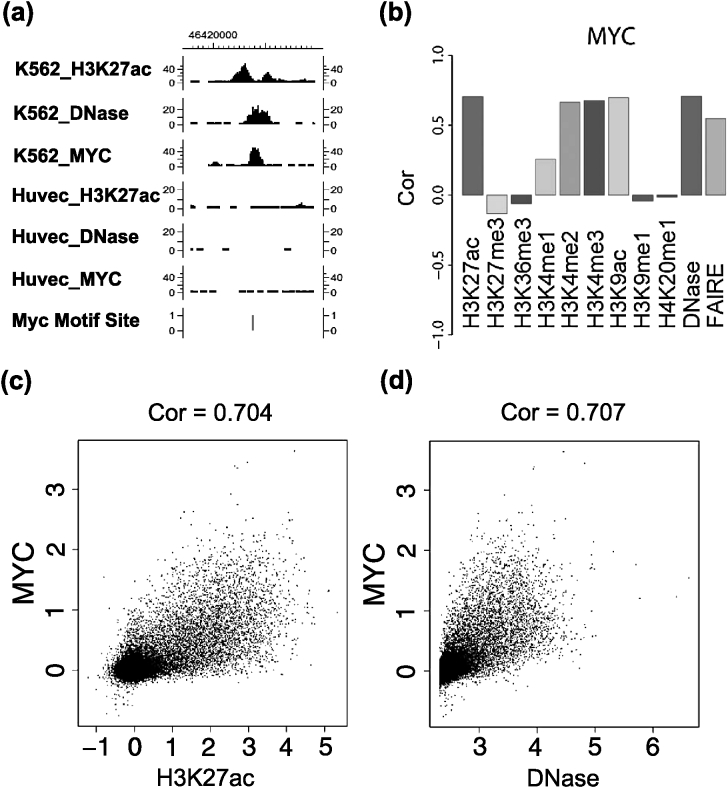
Correlation between TF binding and chromatin features. (**a**) Histone modification H3K27ac ChIP-seq and DNase-seq profiles at a MYC motif site are shown along with ChIP-seq data for TF MYC in two cell lines K562 and Huvec. The profiles shown are read counts in 100-bp sliding windows at 25-bp resolution. MYC binding (i.e., the peak in MYC ChIP-seq data) can be inferred from the H3K27ac and DNase data. In this example, the motif site is bound by MYC in the K562 cell line but not in the Huvec cell line. The cell-type specific binding is correlated with the cell-type specific H3K27ac and DNase I hypersensitivity. In the K562_H3K27ac track, MYC binding leads to nucleosome displacement. As a result, the binding site is surrounded by two nucleosomes carrying the H3K27ac signals [14], causing the dip shape in the signal curve. In the K562_DNase track, the peak reflects the chromatin accessibility due to TF binding. (**b**) Pearson correlation coefficients between different types of chromatin data and the actual MYC ChIP-seq binding intensities in K562 across all MYC motif sites. Certain chromatin features (e.g., H3K27ac, H3K4me2, H3K4me3, H3K9ac, DNase and FAIRE) clearly correlate with MYC binding. (**c**) *A scatter plot* demonstrating the correlation between H3K27ac and MYC ChIP-seq binding intensities in K562 across all MYC motif sites. *Each dot* is a motif site. The binding intensities are normalized and log2-transformed read counts (see Online Resource Supplemental Method 1). ‘Cor’: Pearson correlation coefficient. (**d**) Correlation between DNase-seq and MYC ChIP-seq binding intensities in K562

Computational predictions based on mapping DNA sequence motifs to genome sequences offer an alternative approach to analyze TFBSs [18, 19, 33, 34]. Predictions based purely on DNA sequences, however, are known to have low specificity. In addition, in vivo TF binding is highly context-dependent. Without further information, computationally determined motif sites cannot describe the highly dynamic TF binding activities in different cell types and conditions. Recent technological advances have made it possible to analyze genome-wide chromatin profiles [2, 4, 10, 11, 14–16, 25, 32]. For example, a variety of histone modifications (HMs) (e.g., H3K27ac, H3K4me1, H3K4me2, H3K4me3) can now be measured by ChIP-seq [2, 11, 14, 15]. Additionally, DNase-seq and FAIRE-seq have been developed for mapping DNase I hypersensitivity (DHS) and open chromatin [4, 13, 32]. Analyses of data generated by these technologies show that many chromatin features correlate with TF binding (Fig. 1). As a result, HM ChIP-seq, DNase-seq and FAIRE-seq can serve as a surrogate in place of TF ChIPx for mapping TFBSs [5, 8, 26, 36, 38]. Coupling analyses of these surrogate data with computationally determined motif sites allows one to predict in vivo TF binding. This predictive approach has several unique advantages. First, the requirement for antibodies is easier to satisfy, because ChIP-quality antibodies are available for many HMs, and DNase-seq and FAIRE-seq do not require TF-specific antibodies. Second, measurements offered by HM ChIP-seq, DNase-seq and FAIRE-seq are context-dependent, hence TFBS predictions based on these data are specific to the biological contexts in question (Fig. 1(a)). Third, this approach makes analysis of TFs high-throughput. Among the approximately 1400 human TFs, sequence-specific DNA binding motifs have been determined for about 500 TFs by high-throughput means such as protein microarrays [17, 28, 30, 37, 39]. Thus, the predictive approach allows one to infer TFBSs for hundreds of different TFs simultaneously in one assay. For these reasons, predicting TFBSs based on sequencing chromatin surrogates offers a promising new solution to the global analysis of gene regulation.

As a new approach, many open issues remain to be addressed. Examples include what principles to follow when designing experiments, which guidelines to use to choose informative surrogate data types, and what methods will analyze the data optimally. For statisticians and computational scientists, it is of interest to know what are the crucial analytical challenges and opportunities for developing new methodology. The purpose of this article is twofold. First, through an analysis of the ENCODE data, we will demonstrate some basic characteristics of this approach which will shed light on several important experimental design and data analysis issues. Second, we will use the data to introduce several analytical challenges to investigators who are interested in exploring this new field. For some of these open problems, we will provide our own perspective on potential solutions.

## Key Questions

Our analyses were designed to shed light on the following questions.

1. *Overall prediction performance:* What is the overall accuracy and sensitivity for predicting TFBSs by using chromatin surrogates?
2. *Best surrogate data type:* Which surrogate data type(s), individually or in combination, can produce the best prediction performance?
3. *Supervised versus unsupervised learning:* Predictions can be made by two different approaches. In the unsupervised approach, only surrogate chromatin data are collected. The TFBSs are then predicted based on analyzing the surrogate data at the DNA motif sites. In the supervised approach, one collects ChIP-seq data for at least one TF in addition to generating the surrogate chromatin data. One then uses these data to train a model to predict TFBSs based on the surrogate data. The trained model will be applied to predict binding sites of all other TFs. The supervised approach seems to use more information and intuitively should outperform the unsupervised approach. Is this true? Should one use the supervised approach or the unsupervised one? For the supervised approach, is it possible to eliminate the need for generating the training TF ChIP-seq data by coupling one’s own surrogate data with TF ChIP-seq data from other labs (e.g., existing data in public databases) to train a model, and then apply the model to make predictions?
4. *Possibilities to improve motif-site-based predictions:* Many TFs do not have known motifs and even for the TFs with known motifs, TFBSs may not always occur at the canonical motif sites. Usually a large number of motif sites are found per TF motif, but only a small fraction of the motif sites are actually bound. Thus, it becomes difficult to maintain a low false discovery rate (FDR) without severely reducing statistical power to discover true TFBSs. Is it possible to overcome these limitations?
5. *One-motif-multiple TFs:* What will happen if multiple TFs can recognize a common motif?
6. *From binding sites to functional targets:* One TF may have thousands of predicted binding sites. What fraction of them is functional, in the sense that perturbing the TF expression will result in changes in target gene expression? Is it possible to predict functional target genes?

Answers to these questions have important implications to future studies. For example, answers to (1)–(3) may help one to design future experiments to better allocate available resources. Answers to (1) and (3)–(6) may help statisticians and computational biologists to decide where to invest their efforts for developing the most needed analytical tools.

## Data

To answer these questions, we have analyzed 11 different surrogate data types (Table 1), and constructed various models to predict binding sites of 9 different TFs (Table 2). These data were generated by 6 different labs in the ENCODE consortium and involved two different cell lines for which rich data are available: K562 and Gm12878. The data analyzed represent those available to us from ENCODE at the time the study was initiated, and only TFs with known DNA binding motifs were considered.

**Table 1:** Summary of surrogate chromatin data

Lab	Data type	K562	Gm12878	Description
Broad	H3K27ac	√	√	acetylation of H3 Lysine 27
Broad	H3K27me3	√	√	trimethylation of H3 Lysine 27
Broad	H3K36me3	√	√	trimethylation of H3 Lysine 36
Broad	H3K4me1	√	√	monomethylation of H3 Lysine 4
Broad	H3K4me2	√	√	dimethylation of H3 Lysine 4
Broad	H3K4me3	√	√	trimethylation of H3 Lysine 4
Broad	H3K9ac	√	√	acetylation of H3 Lysine 9
Broad	H3K9me1	√		monomethylation of H3 Lysine 9
Broad	H4K20me1	√	√	monomethylation of H4 Lysine 20
Duke	DNase (DHS)	√	√	DNase I hypersensitivity
UNC	FAIRE	√	√	nucleosome-depleted regions

Available HM ChIP-seq, DNase-seq and FAIRE-seq data in the ENCODE consortium for cell lines K562 and Gm12878 were analyzed. Each row is a data set containing 1–3 replicate samples

**Table 2:** Summary of TF ChIP-seq data

Lab	TF	TF type	K562	Gm12878
HudsonAlpha (HA)	EGR1	activator	√	√
HudsonAlpha (HA)	GABP	activator	√	√
HudsonAlpha (HA)	SRF	activator	√	√
HudsonAlpha (HA)	USF	activator	√	√
HudsonAlpha (HA)	NRSF	repressor	√	√
Yale	E2F4	activator	√	
Yale	E2F6	activator	√	
UTA	MYC	activator	√	√
UTA	CTCF	insulator	√	√

We analyzed 9 different TFs from 3 different labs in the ENCODE consortium for cell lines K562 and Gm12878. Each row is a data set containing 1–3 replicate samples

Nine of the eleven surrogates are histone modifications. Among them, H3K27ac, H3K4me1, H3K4me2, H3K4me3 and H3K9ac correlate with active promoters or enhancers, whereas H3K27me3 is a mark for gene repression [2, 15, 35]. H3K36me3 is enriched in the gene body of actively transcribed genes [2]. H4K20me1 and H3K9me1 have been previously linked to repressive chromatin [31], but recent studies also found correlation between these two HMs with active transcription [2]. As the current understanding of HM functions is incomplete, it is possible that some HMs individually or in combination have unknown new functions. Besides these nine HMs, our surrogates also included DNase I hypersensitivity measured by DNase-seq, which is a signature for DNA binding by trans-acting factors in place of canonical nucleosomes, and open chromatin measured by FAIRE-seq, which is a mark for nucleosome-depleted regions. Among the nine TFs considered, NRSF is a repressor that inactivates neuronal gene transcription in non-neuronal cells. CTCF is a protein that binds to insulators and may also serve as a transcriptional repressor. The other TFs all have roles in activating gene expression.

Since analyses of the two cell lines have reached essentially the same conclusions, this paper will use K562 as an example to demonstrate the main results. In addition to K562 and Gm12878, we have also analyzed DNase-seq and ChIP-seq data for MYC in HelaS3 cells in Sect. 11 to investigate issues related to inferring functional target genes.

## Which Surrogates Are Informative Predictors Individually?

We first investigated which surrogates (i.e., DHS, FAIRE, and various HMs) are most informative for predicting TFBSs. We downloaded aligned ChIP-seq, DNase-seq and FAIRE-seq reads (human genome build 36/hg18) from the ENCODE website (http://genome.ucsc.edu/ENCODE/). Consider *J* surrogate data sets. To predict binding sites of a TF, the DNA binding motif of the TF was mapped to human genome by CisGenome [20] using the default parameters. For each motif site *s* and surrogate data set *j*, the normalized read count *x*_*sj*_ in a 500-bp flanking window centered at the motif site was obtained to represent the surrogate signal intensity (see Online Resource Supplemental Method 1). For each motif site, the actual TF binding intensity *y*_*s*_ was also computed using the ENCODE ChIP-seq data for the TF (Online Resource Supplemental Method 1). We used the surrogate signal intensities *x*_*sj*_ to rank-order motif sites. Top ranked sites were predicted to be bound by the TF. We varied the cutoff and evaluated the predictions using the actual TF binding intensities *y*_*s*_. For evaluation, motif sites with *y*_*s*_>1 were treated as true binding sites. Intuitively, *y*_*s*_>1 means the log2 ratio between the normalized ChIP and Input control read counts is bigger than one (or 2-fold enrichment). Using these as gold standard, we obtained a curve for each surrogate data type that describes the positive predictive values (PPV, i.e., the percentage of true positives among top predictions) at varying cutoffs. We also computed the area under the receiver operating characteristic curve (AUC) for each surrogate and compared different surrogates in terms of AUC.

**Fig. 2:**
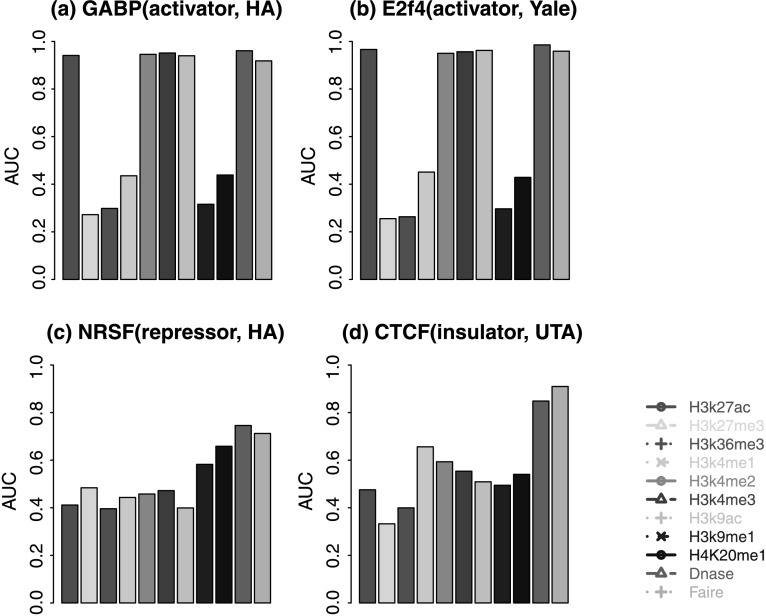
Area under the receiver operating characteristic curves for predicting TFBSs in K562 based on single surrogate. (**a**) GABP; (**b**) E2F4; (**c**) NRSF; (**d**) CTCF. Results for other TFs are in Online Resource Supplemental Fig. S1

When each surrogate was used individually as the predictor, DHS performed the best in most situations based on the global PPV curves and AUC (Fig. 2, Online Resource Supplemental Figs. S1, S2, Supplemental Table S1). Only for CTCF, FAIRE outperformed DHS. Several HMs, including H3K27ac, H3K4me2, H3K4me3 and H3K9ac, also performed well in most but not all data sets. In general, the predictive power of HMs depends on the TF. H3K27ac, H3K4me2, H3K4me3 and H3K9ac predicted TFBSs well for EGR1, GABP, SRF, USF, E2F4, E2F6 and MYC (Fig. 2, Online Resource Supplemental Figs. S1, S2). However, for NRSF, H4K20me1 and H3K9me1 performed better than the other HMs. For CTCF, H3K4me1 performed the best among the tested HMs. These results are consistent with the patterns we saw in Online Resource Supplemental Fig. S3 where Pearson correlation coefficients between the predictors *x*_*sj*_ and the actual binding *y*_*s*_ are compared.

**Fig. 3:**
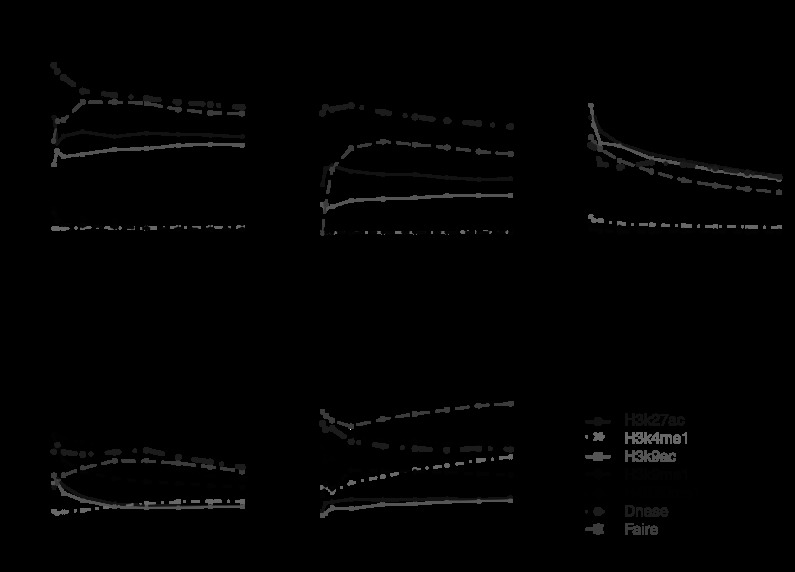
Positive predictive value curves for predicting TFBSs in K562 based on single surrogate. The *x*-axis is the number of the top ranked motif sites. The *y*-axis is the positive predictive value. (**a**) GABP; (**b**) E2F4; (**c**) MYC; (**d**) NRSF; (**e**) CTCF. Only representative surrogates and TFs are shown. See Online Resource Supplemental Fig. S4 for comprehensive results

The above analysis compares the prediction performance globally based on all motif sites. We also examined the PPVs for the top ranked predictions which are most likely to be picked up for follow-up experimental studies (Fig. 3, Online Resource Supplemental Fig. S4). While DHS still performed the best in most data sets, we found a few cases where other surrogates predicted TFBSs better than DHS among the top predictions. For example, for MYC (i.e., c-Myc), H3K27ac and H3K9ac performed better. For NRSF, H4K20me1 outperformed DHS for top 800 motif sites. For CTCF, FAIRE and H4K20me1 performed the best.

In summary, we found DNase I hypersensitivity to be the most consistently accurate predictor for TFBSs, whereas the predictive power of HMs depends on the TF-of-interest.

## How Do Surrogates Perform Jointly?

Next, we asked whether using multiple surrogates together can improve prediction. Let **x**_*s*_=(*x*_*s*1_,…,*x*_*sJ*_)^*T*^ be the vector that contains all surrogate intensities at motif site *s*; we constructed models that use **x**_*s*_ to predict *y*_*s*_.

Before constructing any model, we first investigated whether binding sites of each TF fall into different classes exhibiting different chromatin patterns. For each TF, we clustered its bound motif sites (i.e., sites for which *y*_*s*_>1) based on **x**_*s*_. It turns out that for the same TF, most motif sites bound by the TF share a similar pattern in **x**_*s*_ (Online Resource Supplemental Fig. S5(a)). Next, for each TF, we asked whether its motif sites have regionalized patterns of **x**_*s*_. In this regard, we clustered all motif sites of the TF based on **x**_*s*_ (Online Resource Supplemental Fig. S5(b)). We then examined whether the distribution of the motif sites in each cluster is concentrated on certain genomic regions. However, we did not observe such a phenomenon (Online Resource Supplemental Fig. S5(c), (d)). Furthermore, we checked the correlation between *y*_*s*_ and each surrogate in each chromosome. We found that the correlation patterns in different chromosomes were similar (Online Resource Supplemental Figure S6). Based on these explorations and due to considerations of computational efficiency, we decided not to construct regionalized prediction models with varying forms or parameters for different genomic regions. Instead, for each TF, we constructed models whose form and parameters remain the same across the genome.

Eight prediction methods were tested, including one unsupervised approach and seven supervised learning methods (Table 3; Online Resource Supplemental Method 2). The methods employed include both linear and non-linear models. In the unsupervised approach, the first principal component (PC1) of **x**_*s*_ was computed using all motif sites. The motif sites were then rank-ordered based on PC1. Since the direction of unique PCs can only be determined up to a positive or negative sign, motif sites were sorted based on PC1 and −PC1 separately. Both rankings were tested, and the one with better prediction performance was reported. In the supervised approach, the prediction model was trained using ChIP-seq data for one TF and then applied to other TFs to make predictions. The training methods include linear regression (L) using all surrogates (AS) as predictors, principal component regression (PCR) using the first two principal components of **x**_*s*_s, classification and regression tree (CART), random forest (RF), and support vector regression with linear (SVR_L) and Gaussian (SVR_G) kernels. For the linear regression, we also enumerated all combinations of multiple surrogates (MS), identified the best subset of surrogates using the Mallows’ Cp statistic, and then obtained the linear model based on the best surrogate set (MS_L). For the non-linear models, we did not analyze different surrogate combinations since it would require a tremendous amount of computation time.

**Table 3:** Methods used for prediction

Abbreviation	Category	Description
SS	unsupervised	Single surrogate
AS_PC1	unsupervised	All surrogates, the first principal component
MS_L	supervised	The best subset of surrogates, linear regression
AS_L	supervised	All surrogates, linear regression
AS_PCR	supervised	All surrogates, principal component regression
AS_CART	supervised	All surrogates, classification and regression tree
AS_RF	supervised	All surrogates, random forest
AS_SVR_L	supervised	All surrogates, linear kernel support vector regression
AS_SVR_G	supervised	All surrogates, Gaussian kernel support vector regression

**Fig. 4:**
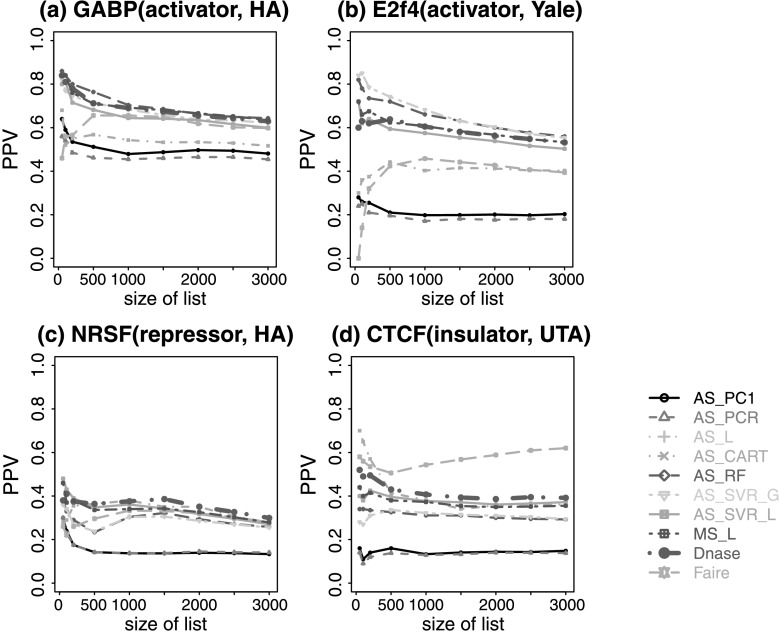
Positive predictive value curves for predicting TFBSs in K562 based on models trained using EGR1. (**a**) Prediction for GABP; (**b**) prediction for E2F4; (**c**) prediction for NRSF; (**d**) prediction for CTCF. Prediction results for other TFs are in Online Resource Supplemental Fig. S7. Using other TFs to train the model produced similar results (data not shown)

Interestingly, we found that even though the best methods based on multiple or all surrogates improved predictions for some TFs compared to analyses based on DHS alone, none of these methods consistently outperformed DHS for all test TFs (Fig. 4, Online Resource Supplemental Fig. S7). For instance, RF and SVR_G trained using EGR1 ChIP-seq data and all surrogates outperformed DHS for E2F4 and E2F6, but performed worse than DHS for NRSF and CTCF. A recent study based on an unsupervised approach has reported that adding HM ChIP-seq did not improve the power for predicting TFBSs using DHS [26]. Our results are consistent with that observation. Differently from [26], however, our analyses here also examined a number of supervised learning approaches. The analyses show that integrating multiple surrogates by these supervised approaches did not improve predictions consistently.

## Supervised Versus Unsupervised Learning

Ranking motif sites based on DHS alone is essentially an unsupervised approach. Figure 4 and Supplemental Fig. S7 show that when DHS is included in the predictors, the gain of using all surrogates and supervised learning over this simple unsupervised method is not universally guaranteed. We speculate that part of the reason is that the supervised approach trains models using one TF and applies it to another TF. Due to intrinsic differences between TFs, the model may be optimized for the training TF but may not be optimal for the test TF. To examine whether this is the case, we compared two prediction scenarios. In scenario 1, a prediction model was trained using surrogate and ChIP-seq data for TF A in a subset of chromosomes (chromosomes 1–16). The model was then applied to predict binding sites of TF A in other chromosomes (chromosomes 17–22 and X). The prediction performance was evaluated using ChIP-seq data for TF A in the test chromosomes (Online Resource Supplemental Figs. S8, S9). In scenario 2, a prediction model was trained using ChIP-seq data for TF A, and then applied to predict binding sites of TF B. The prediction performance was evaluated using ChIP-seq for TF B (Fig. 4, Online Resource Supplemental Fig. S7). In scenario 1, the prediction model trained using AS_L, MS_L, AS_CART, AS_RF, AS_SVR_L and AS_SVR_G all performed better than using DHS alone, and supervised learning on average performed better than unsupervised approaches. In contrast, in scenario 2, supervised prediction based on all surrogates did not consistently outperform DHS (e.g., compare NRSF and CTCF in Fig. 4 and Online Resource Supplemental Fig. S8). This demonstrates that supervised learning was able to improve the prediction for the training TF but cannot guarantee an improvement when the trained model is applied to another TF.

**Fig. 5:**
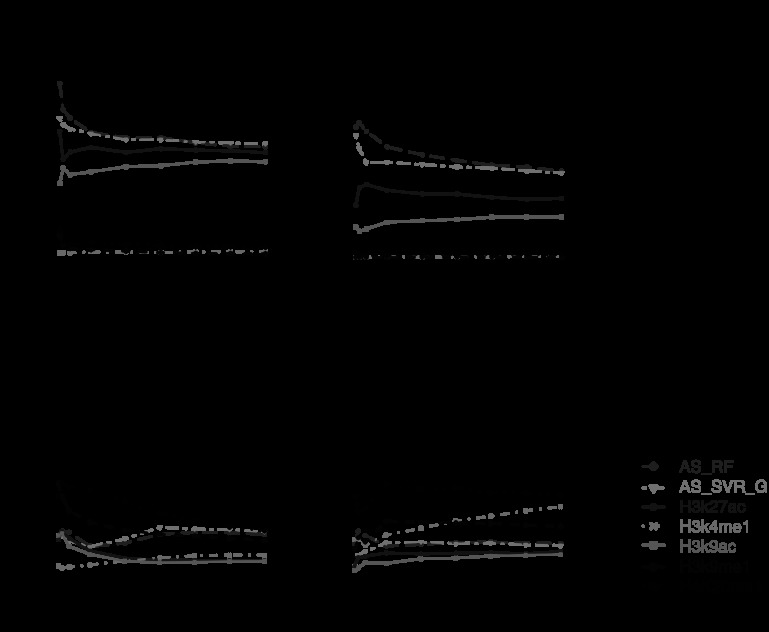
Positive predictive value curves for predicting TFBSs in K562 based on models trained on EGR1 using only HM ChIP-seq data. (**a**) Prediction for GABP; (**b**) prediction for E2F4; (**c**) prediction for NRSF; (**d**) prediction for CTCF. Only representative methods and TFs are shown. See Online Resource Supplemental Fig. S10 for comprehensive results

An investigator may decide to collect HM ChIP-seq data without DNase-seq for other considerations (e.g., if one is primarily interested in studying HMs and the budget does not allow additional DNase-seq). With only HMs as predictors, we observed similar phenomena, that is, the supervised approach did not consistently outperform the unsupervised approach (Fig. 5, Online Resource Supplemental Fig. S10). However, the difference in prediction accuracy between the best supervised method and the best unsupervised method became much bigger. For instance, RF and SVR_G trained using EGR1 ChIP-seq data now performed substantially better than the best unsupervised ranking based on H3K27ac for predicting GABP, SRF, USF, E2F4 and E2F6. For predicting NRSF and CTCF, RF and SVR_G trained by EGR1 performed substantially worse than unsupervised rankings based on H4K20me1.

**Fig. 6:**
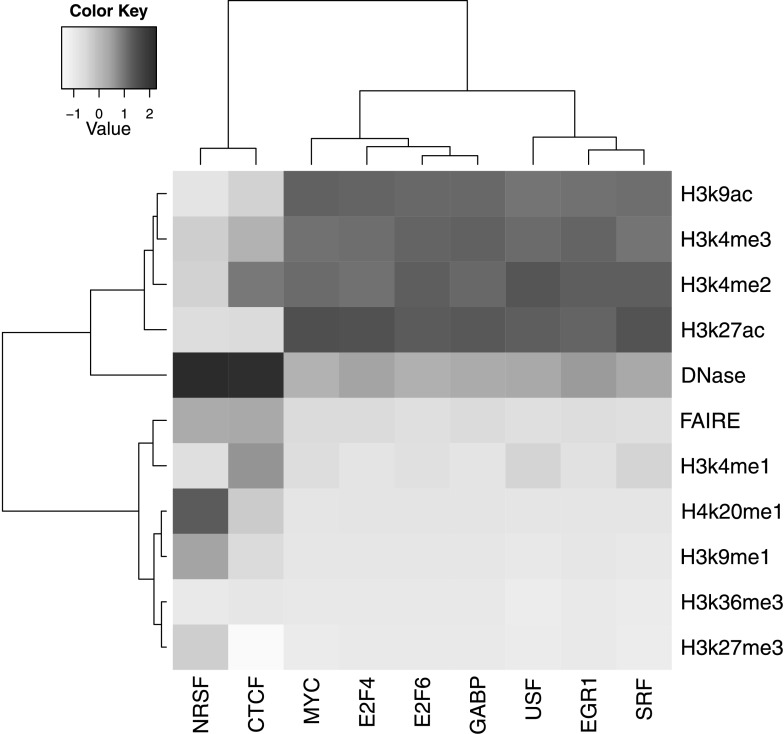
Hierarchical clustering of TFs and surrogates based on the enrichment of the surrogate signals in the bound motif sites compared to the signals in the non-bound motif sites

To further shed light on when the supervised methods can outperform the unsupervised methods, we clustered the nine TFs based on the eleven surrogates. For each TF, the TF’s ChIP-seq data was used to group motif sites into two classes: bound (*y*_*s*_>1) and not bound (*y*_*s*_≤1). The enrichment of the surrogate signals in the bound class compared to the non-bound class was used to cluster TFs (Online Resource Supplemental Method 3). The TFs fall into two distinct classes (Fig. 6). The repressor and insulator proteins NRSF and CTCF were clearly separated from the other TFs which can serve as transcriptional activators. H3K9ac, H3K4me2, H3K4me3 and H3K27ac were clearly enriched in the bound motif sites for those activators but not for NRSF and CTCF.

A careful examination of Figs. 4, 5 and 6 reveals that whether or not the supervised approach improves the unsupervised approach depends on whether or not the training and test TFs are of similar types. For instance, supervised models trained using EGR1 predicted GABP and E2F4 well as they are in the same class (also see SRF, USF, E2F6 in Supplemental Figs. S7, S10), but they did not perform so well for NRSF and CTCF. Interestingly, when we attempted to predict CTCF using models trained by NRSF and only using HMs as predictors, or predict NRSF using models trained by CTCF, supervised learning improved the prediction performance a lot in both cases, compared to predictions based on DHS and FAIRE (Online Resource Supplemental Fig. S11).

Figure 6 shows that DHS is enriched in bound motif sites for all TFs, consistent with the observation that it is the most consistently accurate predictor for all analyzed TFs. This also explains why we observed bigger differences between the best supervised prediction and the best single surrogate based ranking in Fig. 5 after excluding DHS from the predictors, compared with Fig. 4 in which DHS was included as a predictor.

Together, our results suggest that the intrinsic differences among TFs are an important reason why supervised learning based on all surrogates does not guarantee a gain over the unsupervised ranking based on DHS alone. Therefore, when developing future supervised learning methods for predicting TFBSs using surrogate data, it is important to consider the heterogeneity of the TFs. One may need to group TFs into different categories (e.g., activators, repressors, etc.) so that TFs within each category have similar characteristics. One could then train a model for each category in order to take the full advantage of the supervised learning, which may eventually lead to improved prediction accuracy.

## Cross-Lab Prediction

**Fig. 7:**
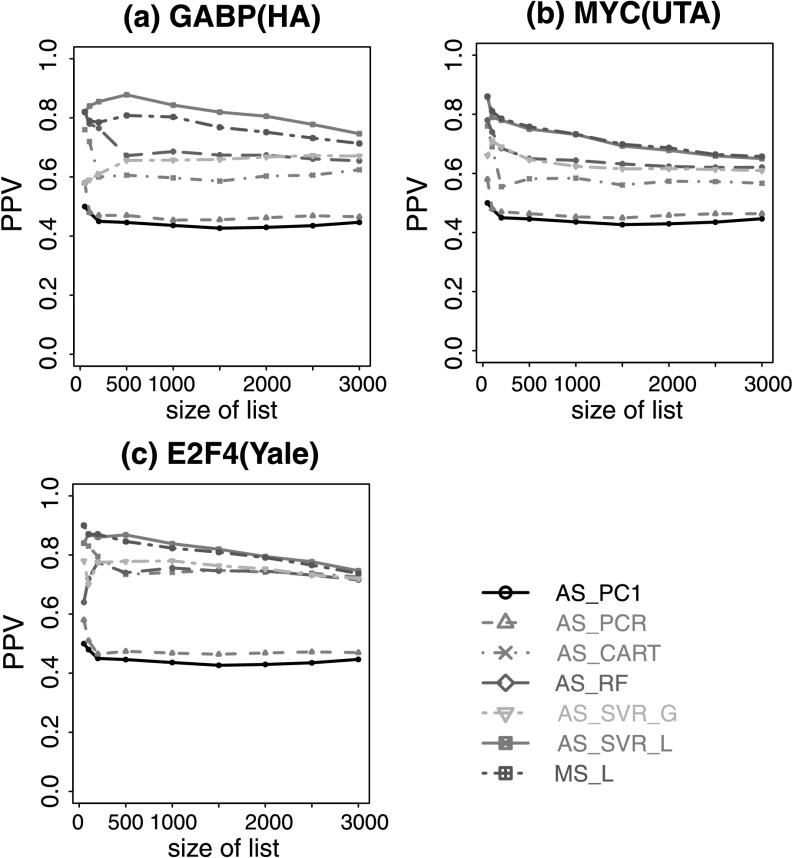
Positive predictive value curves for prediction on EGR1 by models trained using ChIP-seq data from different labs. (**a**) Models trained using GABP (HudsonAlpha); (**b**) models trained using MYC (UTA); (**c**) models trained using E2F4 (Yale). Only representative methods and training TFs are shown. See Online Resource Supplemental Fig. S12 for comprehensive results

Both unsupervised and supervised approaches require one to generate surrogate data for the cell type of interest. For the supervised approach, one also needs to collect training TF ChIP-seq data for different TF classes. If TF ChIP-seq data for the same cell type are available in public databases, a natural question is whether one can couple these public TF ChIP-seq data (typically generated by a different lab) with his/her own surrogate data to train the prediction model, thereby eliminating the needs for generating one’s own TF ChIP-seq. In our analyses, EGR1, GABP and NRSF came from one lab. E2F4 and E2F6 came from another lab. Figures 4, 5 and Supplemental Figs. S7 and S10 show that using the random forest and support vector regression trained by EGR1, one achieved comparable or better prediction performance for predicting E2F4 and E2F6 as compared to predicting GABP and NRSF. Furthermore, when we attempted to predict binding sites for EGR1 by models trained using data from different labs, including USF (HudsonAlpha), SRF (HudsonAlpha), GABP (HudsonAlpha), MYC (UTA), E2F4 (Yale) and E2F6 (Yale), models trained by data from different labs performed similarly (Fig. 7, Online Resource Supplemental Fig. S12). Collectively, these suggest that cross-lab training is feasible, and as ChIP-seq data in public domains continue to grow rapidly, the need to generate one’s own training TF ChIP-seq data may be partially eliminated in the future.

## Sensitivity

**Fig. 8:**
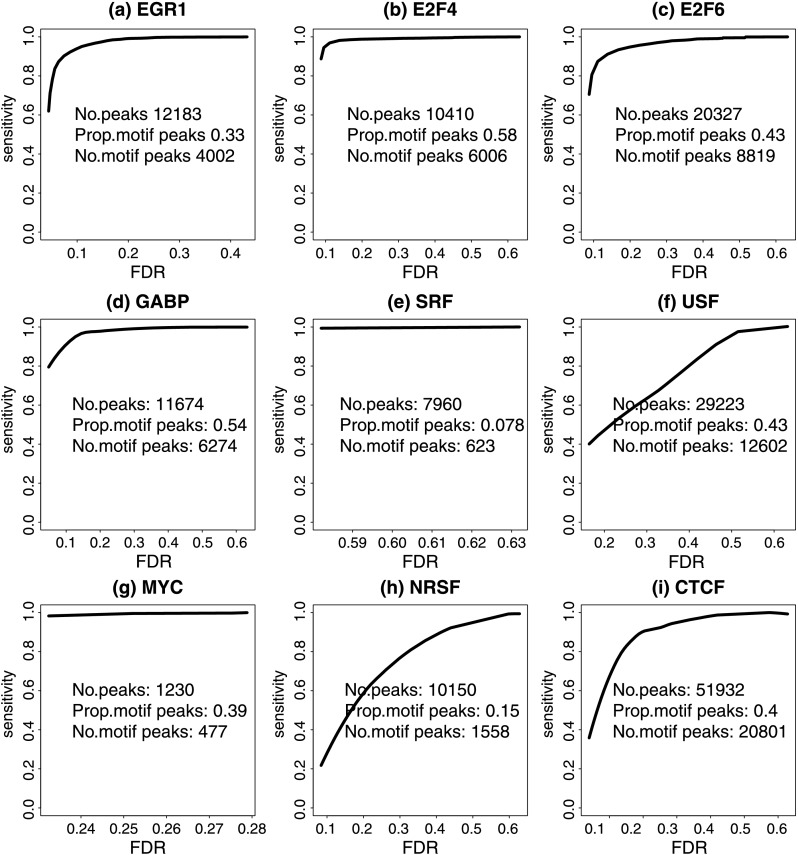
Sensitivity against FDR plot. The *x*-axis is the FDR of DHS at candidate sites. The *y*-axis is the percentage of gold standard motif peaks discovered. “No. peaks” is the total number of gold standard peaks called by CisGenome at FDR 1 %. “Prop. motif peaks” is the proportion of gold standard peaks containing motif sites, called as motif peaks. “No. motif peaks” is the total number of motif peaks. (**a**) EGR1; (**b**) E2F4; (**c**) E2F6; (**d**) GABP; (**e**) SRF; (**f**) USF; (**g**) MYC; (**h**) NRSF; (**i**) CTCF

Since DHS has robustly performed among the best, our subsequent analyses were focused on DHS. To evaluate sensitivity, we analyzed ChIP-seq data for each TF using CisGenomev2 algorithm [20] and called peaks using 1 % FDR as the cutoff. Peaks that contained the motif of the corresponding TF were used as gold standard. In parallel, we ranked motif sites by DHS, used the top ranked sites to predict TFBSs, and estimated the FDR among the predicted sites by comparing their DHS signal distribution to the DHS signal distribution at randomly chosen genomic loci (Online Resource Supplemental Method 4). The receiver operating characteristics (ROC) in Fig. 8 show that at the 25 % FDR level, the predictions were able to recover 50–90 % of the ChIP-seq peaks containing the motifs. SRF is an exception. For SRF, the data were noisy and the lowest prediction FDR we can obtain was 58 %. In practice, this means that none of the predicted SRF binding sites can be claimed as statistically significant. It should be noted that the ROC will change if peaks and motif sites are called using different cutoffs, or if different motifs are used to make predictions. Therefore Fig. 8 should be interpreted as a rough picture of the sensitivity of the prediction approach.

## Challenge 1: Improving Overall Prediction Performance

All our predictions so far were based on analyzing motif sites, but TF binding does not always occur at the canonical motif sites of a TF. Indeed, for most TFs we analyzed, only 30–60 % of the ChIP-seq peaks contained the canonical motifs (Fig. 8). For SRF and NRSF, the percentages were even lower (7.8 and 15.3 % respectively). Therefore, if ChIP-seq peaks without the canonical motifs were included in the gold standard, then the sensitivity of the motif-site-based predictions would drop to below 60 %. In addition, motif-based predictions have two other limitations. First, approximately 900 out of 1400 human TFs do not have known motifs. Second, a motif can have tens of thousands of sites in the human genome. Typically only a small fraction of these sites are bound. Including the large number of non-bound sites in the analysis increases multiplicity and decreases the statistical power. For these reasons, methods for improving the sensitivity and generality of the computational predictions are clearly needed.

**Fig. 9:**
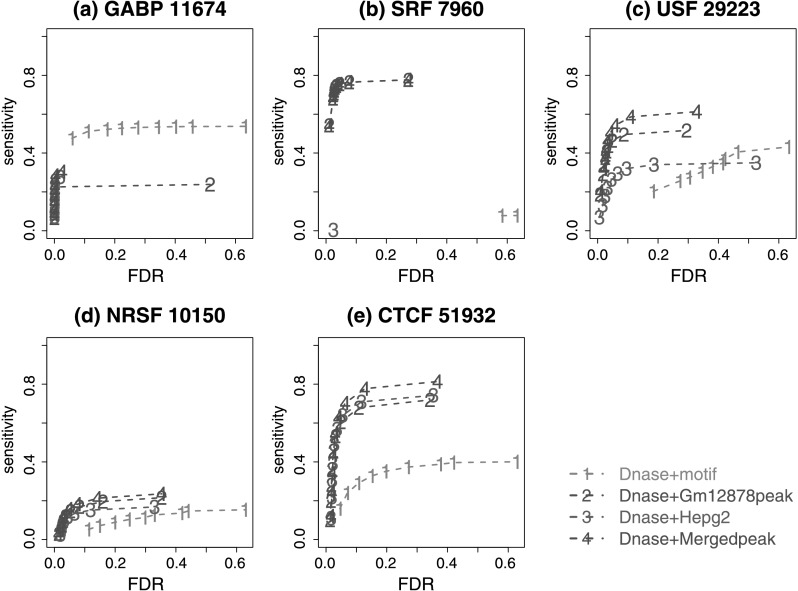
Sensitivity against FDR plot for comparing motif-site-based and existing-peak-based predictions. The *x*-axis is the FDR of DHS at candidate sites. The *y*-axis is the percentage of gold standard peaks discovered. The gold standard peaks are all binding regions called by CisGenome at FDR 1 %, including those without the canonical motifs of the TFs. The number beside the TF name indicates the number of total ChIP-seq peaks. In the existing-peak-based approach, candidate sites were obtained by using ChIP-seq peaks in Hepg2, Gm12878, or their unions

Indeed, there is a big room for improvement. To demonstrate, we have investigated whether one can use experimentally determined TFBSs in existing ChIPx experiments in place of motif sites as candidates for making predictions. For each test TF, we analyzed the ENCODE ChIP-seq data in Hepg2 and Gm12878 cell lines and called peaks using CisGenome at 1 % FDR. The peaks were then merged to serve as candidate sites for making predictions in the K562 cell line. For some TFs, no ChIP-seq data were available for Hepg2. For some other TFs, no peaks were called at the 1 % FDR level in either Hepg2 or Gm12878. These TFs were not included in the analysis. Next, DNase-seq read counts in the K562 cell line were obtained for each candidate site, and the candidates were sorted according to DHS read counts. Finally, to define the gold standard, TF ChIP-seq data in K562 were analyzed, and all peaks, including those that do not contain motifs, were treated as gold standard true binding sites. The ROCs in Fig. 9 indicate that by adopting ChIP-seq peaks from other cell types, better prediction performance was achieved for 4 out of the 5 tested TFs, comparing to the motif-site-based analysis. In some cases, the improvement was substantial. For instance, the sensitivity at 25 % FDR was increased from 20 to 60 % for USF.

Due to cell-type specificity, binding sites from one cell type may fail to characterize binding sites in another cell type. We therefore speculate that by combining ChIP-seq peaks from multiple cell types to derive candidate sites, one may improve the prediction performance. This is confirmed in Fig. 9 which shows that using peaks merged from Hepg2 and Gm12878 as candidates usually performed better than using peaks from each individual cell line. The only case that the motif-based predictions outperformed existing-peak-based predictions was GABP. This might be because GABP peaks in K562 are very different from Hepg2 and Gm12878 peaks. If GABP ChIPx data in more cell types are available, the existing-peak-based analysis may perform better.

Our analyses show that instead of using motif sites, one may use experimentally determined binding sites from other cell types as candidates to make predictions. This approach does not require one to know the TF binding motif, hence the predictions are not limited to motif sites. On the other hand, the power of this approach relies on availability of previous ChIPx data. This highlights the value of publicly available ChIPx data and efforts to compile such data sets. Importantly, ChIPx data in public domains have varying data qualities. In order to effectively utilize these data, methods for measuring data quality, excluding bad quality data sets and incorporating the quality measures into the prediction model, will be needed.

## Challenge 2: One-Motif-Multiple-TF Ambiguity

**Fig. 10:**
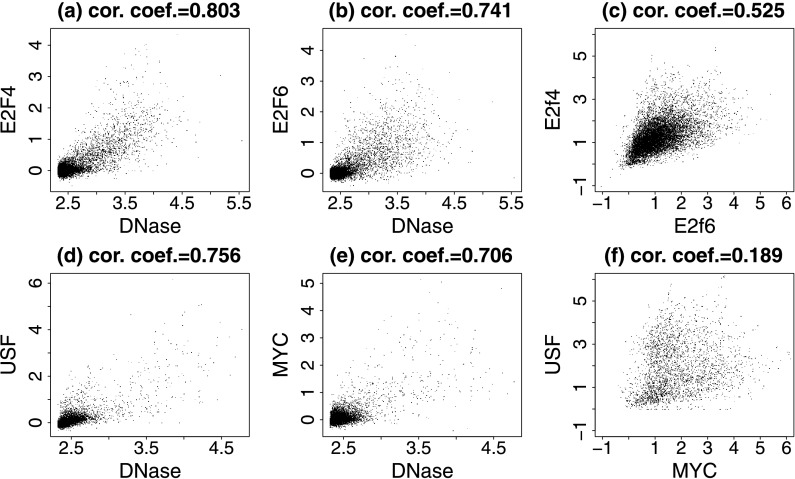
Binding behavior of two TFs recognizing the same motif. (**a**) E2F4 ChIP-seq signal against DNase-seq signal at E2F motif sites; (**b**) E2F6 ChIP-seq against DNase-seq at E2F motif sites; (**c**) E2F4 ChIP-seq against E2F6 ChIP-seq at E2F motif sites with DNase intensities greater than log_2_(10); (**d**) USF ChIP-seq signal against DNase-seq signal at E-box motif sites; (**e**) MYC ChIP-seq against DNase-seq at E-box motif sites; (**f**) USF ChIP-seq against MYC ChIP-seq at E-box motif sites with DNase intensities greater than log_2_(10). All data are from the K562 cell line

Multiple TFs may recognize a common motif. For instance, both MYC and USF can bind to the E-box motif CACGTG, and both E2F4 and E2F6 can bind to the E2F motif TTTCGCGC. When a motif site is predicted to be bound, a natural question is which TF is the one that binds. ENCODE ChIP-seq data for E2F4, E2F6, MYC and USF in K562 allowed us to examine this question. For all four TFs, the ChIP-seq binding intensities at the motif sites correlated with the DHS (Fig. 10(a), (b), (d), (e)). At the motif sites predicted to be bound based on high DHS read count (Online Resource Supplemental Method 5), E2F4 and E2F6 ChIP-seq intensities were highly correlated (Fig. 10(c), cor. coef. = 0.53), whereas the MYC and USF ChIP-seq intensities were only weakly correlated (Fig. 10(f), cor. coef. = 0.19). The correlation between E2F4 and E2F6 suggests that two TFs recognizing the same motif may dynamically compete or cooperate for binding to the same motif sites. The notion that each motif site has a dominant TF that precludes binding of the other TFs may not necessarily be true. On the other hand, in both E2F4-E2F6 and MYC-USF analyses, many motif sites did show preferences for one TF over the other. Whether these differences are purely random or depend on local sequence context and binding of co-factors remain to be explored in the future. If they are not random, then an interesting future research direction will be to develop methods to elucidate the rules for choosing the preferred binding TFs (e.g., by modeling local sequence context or introducing better motif models that discriminate competing TFs).

## Challenge 3: From Binding Targets to Functional Targets

**Fig. 11:**
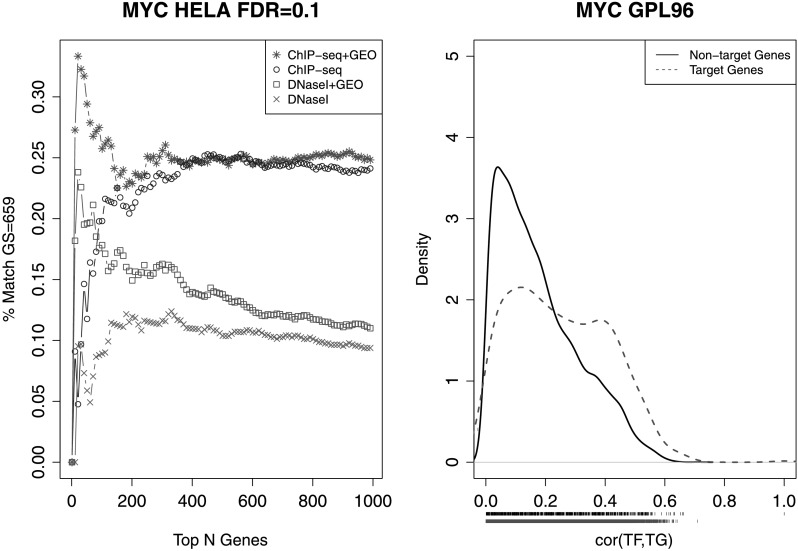
Predicting functional target genes from DHS (DNase I) and GEO databases. (**a**) Prediction accuracy plot for MYC in HelaS3 cells showing the percentage of correctly predicted target genes (*y*-axis) among the top N ranked genes (*x*-axis). Each set of predictions are based on four different data sources: (1) MYC ChIP-seq in HelaS3 cells combined with a compendium of HGU133A samples stored in GEO, (2) MYC ChIP-seq in HelaS3 cells alone, (3) DNase I in HelaS3 cells combined with a compendium of HGU133A samples stored in GEO, and (4) Dnase I in HelaS3 cells alone. There is a clear prediction improvement for both the ChIP-seq predictions and Dnase I predictions when integrating the information contained in the gene expression profiles stored in GEO. (**b**) Density plot of the correlations between each gene and MYC in the HGU133A GEO compendium for MYC HelaS3 target genes (*red dashed line*) and HelaS3 non-target genes (*black solid line*). Observed correlation values are indicated by the ticks below the *x*-axis, while the smoothed density estimate is plotted

In ChIPx experiments, a TF can have tens of thousands of binding sites. Typically only a small fraction of these TFBSs are functional, in the sense that perturbation of the TF expression will change the target gene expression in the biological context in question [12, 22]. Similarly, one can expect that only a fraction of the predicted TFBSs using surrogate data are functional. Among the TFs we tested, we searched the Gene Expression Omnibus (GEO) [1] and found one data set in which gene expression profiles were obtained before and after perturbing MYC expression in HelaS3 cells. For HelaS3, ENCODE has generated DNase-seq and MYC ChIP-seq data. These data allowed us to examine the fraction of DHS-predicted MYC TFBSs that are functional. Functional target genes were defined as genes bound by MYC in promoters based on MYC ChIP-seq and transcriptionally responded to MYC perturbation in the gene expression data (Online Resource Supplemental Method 6). Among the top 1000 genes bound by MYC determined by ChIP-seq, only about 25 % were functional targets (Fig. 11). Among the binding target genes predicted by DNase-seq, only about 10 % were functional targets. These results show that identifying binding sites is only the first step toward elucidating the global regulatory program. Methods for determining functional targets are also needed. Although in theory one can perform gene perturbation experiments for each TF, this approach is expensive and low-throughput with respect to TFs, similar to performing ChIP-seq experiments for each TF. Thus, global prediction of TFBSs will need to be accompanied by a method for global identification of functional target genes, which is an open problem that requires new solutions.

Analysis of large amounts of gene expression data in GEO shows that the rich information in GEO may be used to help with identification of functional targets. If a gene is a target of a TF, one would expect that the expression of the gene and the TF should be positively or negatively correlated across the diverse cell types. To this end, we examined the Pearson correlation between MYC and each individual gene in the human genome using a published compendium of 13,182 Affymetrix HGU133A array samples [24]. The gene expression samples were consistently normalized using the frozen RMA algorithm [23]. This analysis shows that the correlation coefficients for functional targets tend to be bigger than non-target genes (Fig. 11). We ranked the predicted MYC binding targets by DHS and GEO correlation respectively, and took the average of the two ranks to score and re-order MYC binding target genes. The top genes ranked in this way had a much higher proportion of functional targets compared to ranking predicted targets by DHS alone (20 vs. 10 %). Using a similar approach, we also improved functional target prediction based on MYC ChIP-seq. Together, this shows that public gene expression data in GEO, despite their heterogeneity and potential lab and batch effects, do contain valuable information that may be used to distinguish functional and non-functional target genes of a TF. Whether this observation holds true in general and how to optimally use this information remain to be explored in future with other TFs when more triplet data sets consisting of DNase-seq, ChIP-seq, and TF perturbation gene expression data become available. Our present analyses however suggest, that with enormous amounts of public genomics data in public domains, novel statistical and computational methods for integrating different sources of information may play an important role in high-throughput identification of functional target genes of TFs.

## Conclusions and Discussion

Through the analyses of ENCODE data, we have verified that TFBSs can be predicted using chromatin surrogates with reasonable accuracy and sensitivity. This approach offers an attractive alternative to ChIP-seq and ChIP-chip as it allows one to survey many TFs together in one assay. Our analyses show that DNase I hypersensitivity profiled by DNase-seq consistently performed among the best as a predictor, whereas the performance of using a specific HM as the predictor may depend on TFs. Thus if the available resources only allow one to sequence one surrogate data type, one may consider DNase-seq.

When TF ChIP-seq data are available in addition to multiple types of surrogate data, one may choose to use these data to train a prediction model and then apply the model to predict binding sites for other TFs. Our analyses show that the improvement the supervised learning can provide over the unsupervised method is not significant when DHS is included as a predictor. When only HMs are used as predictors, the gain of supervised learning over the unsupervised approach depends on whether the training and test TFs belong to similar classes. If these two TFs have distinct properties (e.g., one is activator, whereas the other one is repressor), then the supervised learning approach may not improve over the unsupervised methods. Therefore, the advantage of supervised learning for HMs only is also not universally guaranteed. Investigators developing such methods may need to develop different prediction models for different TF classes.

A recent study of 203 yeast TFs have shown that yeast TFs fall into two categories: histone-sensitive TFs and histone-insensitive TFs [8]. The target genes of histone-sensitive TFs have relatively higher HM signals and are easier to be predicted using HMs. The histone-sensitive TFs are also more likely to interact with chromatin modifiers and are enriched in the upper layers of regulatory hierarchy. Whether these phenomena hold true in human is an interesting problem. Our results suggest that human TFs are very likely to fall into different categories as well. On the other hand, since we only have data from a limited number of human TFs, including only one repressor NRSF and one insulator binding protein CTCF, and since the knowledge of the TF network in humans is still incomplete as most human TFs do not have ChIP data, we were not able to meaningfully examine the statistical association between different TF categories and their ability to interact with histone modifiers, or their positions in the regulatory hierarchy. These issues are worthwhile to be reexamined in the future as sufficient data would become available.

Our analyses did not use the curve shape information in the surrogate chromatin data. Several studies show that DNase-seq and some HM ChIP-seq profiles have characteristic footprints surrounding TFBSs. For instance, many of these surrogates have a characteristic dip structure around the *bona fide* binding sites (Fig. 1(a)). Incorporating the shape information into the prediction model may further increase the prediction power [5, 26].

Supervised learning requires training TF ChIP-seq data. As more ChIPx data become available in public domains, it may be possible to couple these public data with one’s own surrogate data to train the prediction models. This may allow one to reduce the experimental cost.

Instead of using motif sites as candidates for the analysis, one may also use experimentally determined binding sites of the same TF collected from publicly available ChIPx data sets, typically in other cell types. This existing-peak-based prediction approach is not restricted to motif sites and is applicable to TFs without known motifs. Our results show that it frequently improves the prediction performance over the motif-site-based prediction approach, perhaps also due to elimination of large amounts of motif sites that are never bound by the TF. As more ChIPx data accumulate, the pool of experimentally determined TFBSs will become increasingly more comprehensive, which in turn may increase the power of this approach.

The two applications of public ChIPx data highlights the value of compiling such data. Importantly, methods for assessing data quality are needed to ensure that bad quality data sets will be excluded to avoid misleading supervised learning or candidate site identification. Statistical methods that can integrate the quality measures into the prediction pipeline may also be needed.

Predictions based on DNase-seq and other surrogate data are complementary to ChIPx. ChIPx are still useful to accurately determine direct binding of a TF of interest. When designing future experiments, one may couple DNase-seq for surveying many TFs with relatively low accuracy and sensitivity with ChIP-seq for analyzing selected TFs with high accuracy and sensitivity. With DNase-seq available, one question that remains to be addressed but not discussed in this paper is whether one can reduce the sequencing depth of the ChIP-seq library but still keep similar sensitivity by integrating DNase-seq data into ChIP-seq analysis. If so, this will allow one to reduce the experimental cost, which is particularly useful if one wishes to analyze many TFs using ChIPx in detail, or analyze the same TF in many different developmental time points or biological conditions. For statisticians, this will create a need for new data integration methods.

The observation that DHS alone predicted TFBSs reasonably well seems to suggest that there is no much room for statisticians to develop new methods. However, this is not true if one realizes that predicting TFBSs is not our final goal. It remains unclear how one should resolve the one-motif-multiple-TF ambiguity. Moreover, only a small fraction of binding sites are functional. How to identify the small subset of functional binding targets remains a significant challenge. Our initial analysis shows that public gene expression data in GEO may help, but how to systematically and optimally use this information and information from other genomics data types needs to be further explored. These examples show that research related to predicting TFBSs by sequencing chromatin states is filled with unsolved open problems. Data scientists will find this research to be both challenging and exciting.

## Electronic Supplementary Material

Below is the link to the electronic supplementary material.

Online Resource for Global Mapping of Transcription Factor Binding Sites by Sequencing Chromatin Surrogates: A Perspective on Experimental Design, Data Analysis, and Open Problems (PDF 2.2 MB)
