# Supplementary material for: Global Mapping of Transcription Factor Binding Sites by Sequencing Chromatin Surrogates: a Perspective on Experimental Design, Data Analysis, and Open Problems
Source: Stat Biosci. 2012 May 23;5(1):156–78. doi: 10.1007/s12561-012-9066-5 (PMC3677239; doi:10.1007/s12561-012-9066-5)
Supplement: Supplementary file 1 — Online Resource for Global Mapping of Transcription Factor Binding Sites by Sequencing Chromatin Surrogates: A Perspective on Experimental Design, Data Analysis, and Open Problems (PDF 2.2 MB) [file 12561_2012_9066_MOESM1_ESM.pdf]

Noname manuscript No.
(will be inserted by the editor)

Online Resource for Global Mapping of Transcription Factor Binding Sites by Sequencing Chromatin Surrogates: A Perspective on Experimental Design, Data Analysis, and Open Problems

Yingying Wei · George Wu · Hongkai Ji*

Received: date / Accepted: date

1 Supplemental Method 1: Data preprocessing

In order to predict TF binding based on chromatin surrogates at motif sites, DNA binding motif of each TF was mapped to human genome using CisGenome [4] with default parameter settings. For each TF, the corresponding DNA binding motif was obtained from either TRANSFAC [12] or publication [6]. Consider a single TF. For each motif site s and surrogate dataset j, a normalized read count x_{sj} was computed to represent the signal intensity of HM, DNase I hypersensitivity or open chromatin in a 500bp flanking window centered at the motif site. For each motif site, the actual TF binding intensity y_s was also computed using the ChIP-seq dataset for the TF. Conceptually, our goal is to predict which motif sites are bound by the TF based on x_{sj}s. The predictions can be evaluated based on y_s.

To compute x_{sj} and y_s, sequence reads in each ChIP-seq sample were extended 150bp to 3' end to approximately reconstruct the original DNA fragments. Based on ENCODE annotations, 150bp reflects the most typical DNA fragment length in these samples. After dividing the genome into 10bp non-overlapping bins, the number of DNA fragments covering each bin was counted. Let C_{ijk} be the raw count for bin i, dataset j and replicate k. Let N_{jk} be the total fragment count in sample (j, k). Normalize C_{ijk} by $c_{ijk} = C_{ijk} * min_{(j',k')} N_{j'k'}/N_{jk}$, and transform the normalized value to $b_{ijk} = log_2(\Delta + c_{ijk})$. Δ is an offset added to avoid log(0) and unstable es-

∗ To whom correspondence should be addressed

Yingying Wei · George Wu · Hongkai Ji
Department of Biostatistics, The Johns Hopkins University Bloomberg School of Public Health, 615 North Wolfe Street, Baltimore, MD 21205

Hongkai Ji
Tel.: (410)955-3517
Fax: (410)955-0958
E-mail: hji@jhsph.edu

timate of fold changes when c_{ijk} is small. Different values of Δ (1, 5, and 10) were tried, and they produced the same qualitative conclusions. In the paper, we only show results based on $\Delta = 5$ for simplicity. After obtaining b_{ijk}s, replicates were averaged to obtain $a_{ij} = \sum_k b_{ijk}/n_j$. n_j is the number of replicate samples in dataset j. If control samples were available (e.g., Input controls in ChIP-seq experiments), control read counts were subtracted from the ChIP read counts to obtain $a_{ij} = \sum_k b_{ijk}/n_j - \sum_k b_{ij'k}/n_{j'}$. Here j' indicates the control dataset corresponding to the ChIP dataset j. a_{ij} provides a one number summary for each bin i and dataset j. Next, each motif site was extended 250bp to both ends. The a_{ij}s within the 500bp window were averaged to obtain x_{sj}. y_s was computed similarly.

2 Supplemental Method 2: Various prediction methods

We compared nine different methods for predicting TFBSs.
Unsupervised methods:

1. Single surrogate (SS): TFBSs are predicted based on each individual surrogate. It was assumed that y_s is a monotone function of x_{sj}, and ranking of motif sites based on x_{sj} determines the ranking of motif sites based on y_s. Top ranked motif sites were predicted to be TFBSs.
2. Principal component of all surrogates (AS_PC1): Let $\mathbf{x}_s = (x_{s1}, \ldots, x_{sJ})^T$ be the vector that contains the intensity values of all surrogates at motif site s. The first principal component (PC1) [5] of all $\mathbf{x}_s$s was computed and motif sites were ranked accordingly. Since the direction of unique PCs can only be determined up to a positive or negative sign, motif sites can be ranked based on either PC1 scores or $-1\times$PC1. Both rankings were tested, and the ranking that produced better results was reported.

Supervised methods:

1. Best subset of multiple surrogates, linear regression (MS_L): Using a training TF ChIP-seq dataset, the following linear model $y_s = \beta_0 + \beta_{j_1} x_{sj_1} + \cdots + \beta_{j_k} x_{sj_k} + error$ is fit using a subset of surrogates $(j_1, \cdots, j_k)$. All possible combinations of surrogates were enumerated and tested. The exhaustive search finds the best subset of surrogates using the Mallows' Cp as the selection criterion. The linear model based on the best surrogate combination will be used as the final prediction model to predict TFBSs for other TFs [9].
2. All surrogates, linear regression (AS_L): Using a training TF ChIP-seq dataset, a prediction model $y_s = \beta_0 + \sum_j \beta_j x_{sj} + error$ was fit using all surrogate data types. The trained model was used to predict TFBSs for other TFs.
3. All surrogates, principal component regression (AS_PCR): The first two PCs [5] of $\mathbf{x}_s$s were used as covariates to fit a regression using a training TF ChIP-seq dataset: $y_s = \beta_0 + \beta_1 PC1 + \beta_2 PC2 + error$. The trained model was used to predict TFBSs for other TFs.

4. All surrogates, CART (AS_CART): Using a training TF ChIP-seq dataset and all surrogates, a prediction model was trained using the classification and regression tree algorithm [3],[10], which was then applied to make predictions for new TFs.
5. All surrogates, Random Forest (AS_RF): The prediction model was trained using all surrogates and random forest [2],[8].
6. All surrogates, linear kernel SVR (AS_SVR_L): The prediction model was trained using all surrogates and support vector regression with a linear kernel[3],[7]: $K(\mathbf{x}, \mathbf{x}') =< \mathbf{x}, \mathbf{x}' >$.
7. All surrogates, Gaussian kernel SVR (AS_SVR_G): The prediction model was trained using all surrogates and support vector regression with a Gaussian kernel [3],[7]: $K(\mathbf{x}, \mathbf{x}') = exp(-\gamma \parallel \mathbf{x} - \mathbf{x}' \parallel^2)$

To compare different methods, TF ChIP-seq was used as gold standard. For instance, we train a model using TF A, say EGR1, and use the model to predict binding sites of TF B, say GABP. We can then use TF B ChIP-seq data to benchmark prediction performance. TF B motif sites with $y_s > 1$ were treated as true binding sites. For each prediction method, motif sites were rank ordered based on the predicted TF binding intensities. The positive predictive value (PPV) (i.e., the percentage of true positives among the top predictions) was reported for the top N predictions, where $N = 1, 2, \cdots, etc.$ This created a curve showing the PPV as a function of N. Curves of different methods were compared. The area under the receiver operating characteristic curves (AUC) was also computed and compared across methods. In parallel, we also computed and compared the Pearson correlation between y_s (from the actual TF B ChIP-seq data) and the predicted binding intensities for each prediction model.

3 Supplemental Method 3: Clustering analysis

To generate Figure 6, we first took the average of x_{sj} as computed in Supplemental Method 1 over the TF bound motif sites (where the TF ChIP-seq $y_s > 1$) and the TF non-bound sites (where $y_s \leq 1$) respectively for TF t and surrogate data type j. This created the average surrogate signals for the bound and non-bound sites, denoted by u_{tj} and v_{tj} respectively. Next, we subtracted v_{tj} from u_{tj} to obtain $r_{tj} = u_{tj} - v_{tj}$. Since x_{sj}, u_{tj} and v_{tj} were all on log2 scale, r_{tj} describes the enrichment of the surrogate signals in the bound class compared to the non-bound class for surrogate data type j and TF t. Using r_{tj}s, we then conducted a hierarchical clustering using Euclidean distance and complete linkage, and the result is shown as a heat map.

4 Supplemental Method 4: Sensitivity analysis

To generate Figure 9, ChIP-seq binding regions for each TF in Hepg2 and Gm12878 cell lines were detected using CisGenome [4] at 1% FDR. We recorded

the center of binding regions as peak sites. When combining peak sites from Hepg2 and Gm12878, we merged any two binding regions if the distance between their centers were less than 250bp. Now these peak sites detected in other cell lines can serve the same function as our motif sites, therefore from now on we call both of them candidate sites. Using the mapped DNase-seq reads downloaded from ENCODE, we counted the number of reads in a 500bp flanking window of each candidate site which was used as the predictor.

For Figures 8 and 9, to calculate the false discovery rate (FDR) for each candidate site, we need to learn the null distribution of DNase intensities. For that purpose, we randomly sampled 1,000,000 loci without replacement from the whole genome and used these loci as our null motif sites. Then we counted DNase read numbers for the null motif sites in the same way as before, and obtained the background null distribution $p_0(x)$. For a given TF A and a candidate binding sites list, we ranked sites according to the decreasing order of DNase read count. At each cutoff k of the rank list, we computed the p-value based on the null distribution $p_0(x)$ and estimated the false discovery rate (FDR) using the Benjamini-Hechberg procedure [1].

To compute the sensitivity in Figure 9, we first used CisGenome [4] to call peaks for TF ChIP-seqs in K562, and adopted these peaks as the gold standard. For Figure 8, we used the ChIP-seq peaks containing the canonical motifs as the gold standard. Next, for each FDR cutoff, we detected candidate sites passing the cutoff, and counted the number of gold standard ChIP-seq peaks that were discovered by these sites. Discovery of a gold standard peak is defined as existence of one or more positive motif sites located within 250bp of the center of the peak. Sensitivity (i.e. [Number of ChIP-seq peaks found among the top predictions]/[Total number of ChIP-seq peaks]) of each candidate sites list was computed accordingly.

5 Supplemental Method 5: One-motif-multiple-TFs

We computed E2F4 and E2F6 ChIP-seq binding intensities at 347,952 E2F motif sites using the way described in Supplemental Method 1. The intensities intuitively are log2 ratio of normalized ChIP and Input control read counts, with an offset Δ introduced to the counts. Since antibodies for different TFs may have different efficiencies, the ChIP-seq intensities for E2F4 and E2F6 may not be directly comparable. Therefore, we quantile normalized the E2F4 and E2F6 ChIP-seq intensities and focused on studying their correlation. We plotted the quantile normalized intensities of E2F4 against DHS, and quantile normalized E2F6 intensities against DHS. E2F motif sites with DHS intensity greater than $log_2(10)$ were predicted to be bound by a TF. For these motif sites, we also plotted the quantile normalized intensities of E2F4 against the quantile normalized intensities of E2F6. We repeated the same analysis for USF against MYC at 139,222 E-box motif sites. E-box consensus CACGTG was mapped to human genome without allowing any mismatch. The obtained motif sites were used for comparing USF and MYC.

6 Supplemental Method 6: Functional target analysis

To study functional targets, we first analyzed MYC ENCODE ChIP-seq data from HelaS3 and GEO gene expression data (GSE5823) before and after TF perturbation. ChIP-seq were analyzed using CisGenome [4] with the default settings to call peaks. 3346 peaks corresponding to 2772 unique genes were found ($FDR < 10\%$). Gene expression data were analyzed using limma [11] which identified 3019 differentially expressed genes at FDR = 10%. In total, 659 unique genes had at least one overlapping MYC binding peak in the -25kb $\sim$ +10kb region around the transcription start site (TSS) and was differentially expressed. These genes were defined as the functional targets of MYC in HelaS3.

We then mapped MYC motif to genome and extracted DNase-seq read count for each motif site. For each gene, motif sites within the -25kb $\sim$ +10kb were collected and their DNase I read counts were added up. Genes were then ranked based on the DNase I signal. Figure 11a shows what percentage of top ranked genes are functional targets. Next, for each gene, we also computed its Pearson correlation with MYC across 13,182 gene expression microarray samples in the GEO compendium. Genes were also rank ordered based on the absolute value of the Pearson correlation. The average of the DNase rank and GEO rank was taken as the final score of each gene, and genes were re-ranked accordingly. For this new ranking method, the percentage of top ranked genes that are functional targets is also shown in Figure 11a. Based on the figure, integrating GEO data clearly improved functional target identification.

We performed similar analysis by using MYC ChIP-seq in place of DNase-seq to rank genes. Integrating GEO data again improved.

References

1. Benjamini, Y and Hochberg, Y (1995). Controlling the false discovery rate: a practical and powerful approach to multiple testing., Journal of the Royal Statistical Society, Series B (Methodological) 57, 289-300
2. Breman L (2001) Random Forests. Machine Learning, 45, 5-32
3. Hastie T, Tibshirani R, Friedman J (2002) The elements of statistical learning: data mining, inference, and prediction, second edition. Springer, New York.
4. Ji H, Jiang H, Ma W, Johnson DS, Myers RM, Wong WH (2008) An integrated software system for analyzing ChIP-chip and ChIP-seq data. Nat Biotechnol 26, 1293-1300
5. Jolliffe I (2002) Principal component analysis, second edition. Springer-Verlag, New York.
6. Kim TH, Abdullaev ZK, Smith AD, Ching KA, Loukinov DI, Green RD, Zhang MQ, Lobanenkov VV, Ren B (2007) Analysis of the vertebrate insulator protein CTCF-binding sites in the human genome. Cell 128, 1231-45.
7. R package e1071
8. R package randomForest
9. R package leaps
10. R package rpart
11. Smyth, G (2004) Linear models and empirical bayes methods for assessing differential expression in microarray experiments. Statistical Applications in Genetics and Molecular Biology. 3, Article 3.
12. Wingender E, Dietze P, Karas H, Knuppel R (1996) TRANSFAC: a database on transcription factors and their DNA binding sites. Nucleic Acids Res. 24, 238-41

Table S1 Top ranked surrogate data types based on AUC

TF	rank1	rank2	rank3	rank4	rank5
EGR1	DNase	H3K4me2	H3K4me3	H3K9ac	H3K27ac
GABP	DNase	H3K4me3	H3K4me2	H3K27ac	H3K9ac
SRF	DNase	H3K4me2	H3K4me3	FAIRE	H3K27ac
USF	DNase	H3K4me2	H3K4me3	FAIRE	H3K27ac
E2F4	DNase	H3K27ac	H3K9ac	FAIRE	H3K4me3
E2F6	DNase	H3K4me2	H3K4me3	H3K9ac	H3K27ac
MYC	DNase	H3K27ac	H3K9ac	H3K4me3	H3K4me2
NRSF	DNase	FAIRE	H4K20me1	H3K9me1	H3K27me3
CTCF	FAIRE	DNase	H3K4me1	H3K4me2	H3K4me3

We rank the surrogate data types in terms of AUC for predicting TFBSs of each TF and list the top five surrogate data types.

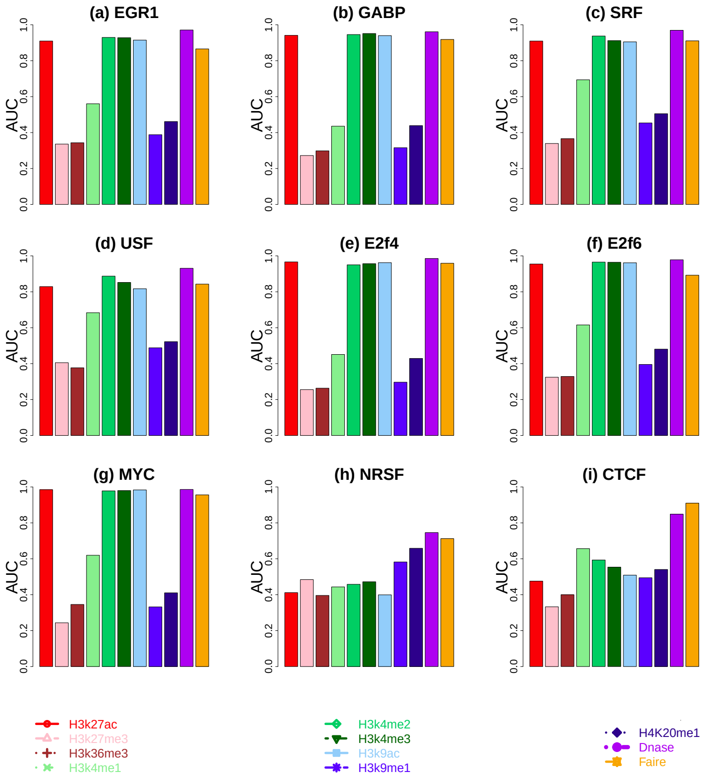

Fig. S1 Area under the receiver operating characteristic curves for predicting TFBSs in K562 based on single surrogate. (a) EGR1; (b) GABP; (c) SRF; (d) USF; (e) E2F4; (f) E2F6; (g) MYC; (h) NRSF; (i) CTCF.

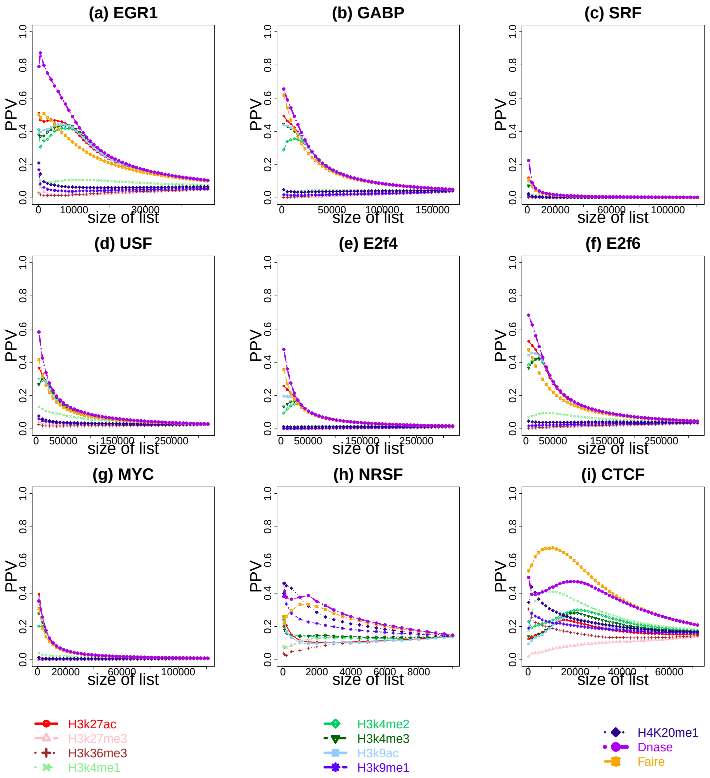

Fig. S2 Positive predictive value curves for predicting TFBSs in K562 based on single surrogate over all motif sites' ranges. The x axis is the number of the top ranked motif sites. The y axis is the positive predictive value, i.e., the percentage of true positives among the top predictions. (a) EGR1; (b) GABP; (c) SRF; (d) USF; (e) E2F4; (f) E2F6; (g) MYC; (h) NRSF; (i) CTCF.

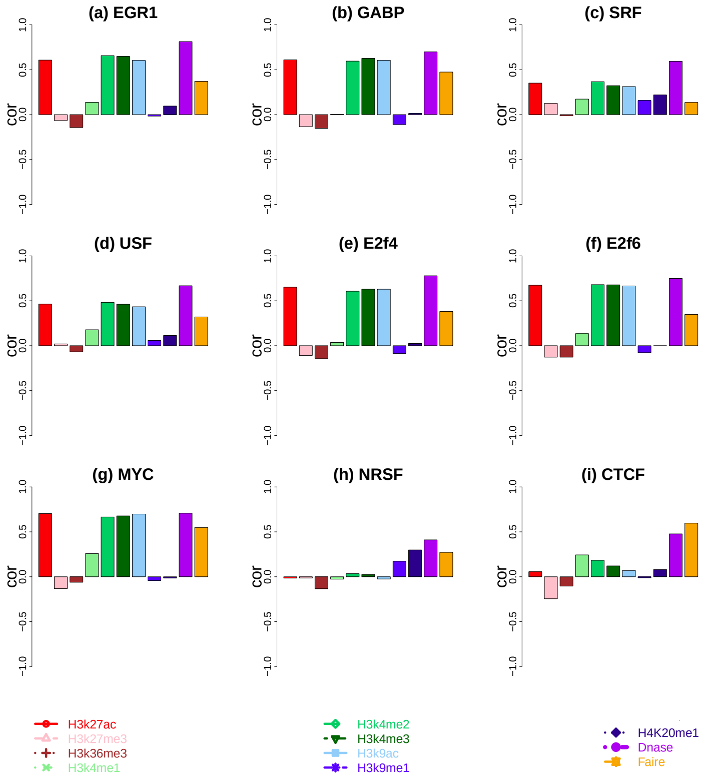

Fig. S3 Pearson correlation coefficients between the predictors and the actual ChIP-seq binding intensity in K562. (a) EGR1; (b) GABP; (c) SRF; (d) USF; (e) E2F4; (f) E2F6; (g) MYC; (h) NRSF; (i) CTCF.

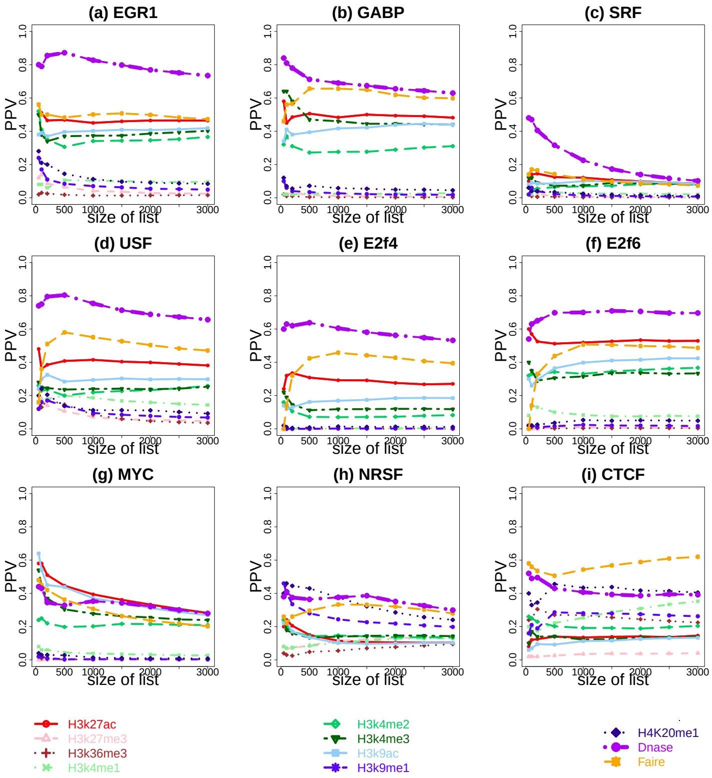

Fig. S4 Positive predictive value curves for predicting TFBSs in K562 based on single surrogate. The x axis is the number of the top ranked motif sites. The y axis is the positive predictive value. (a) EGR1; (b) GABP; (c) SRF; (d) USF; (e) E2F4; (f) E2F6; (g) MYC; (h) NRSF; (i) CTCF.

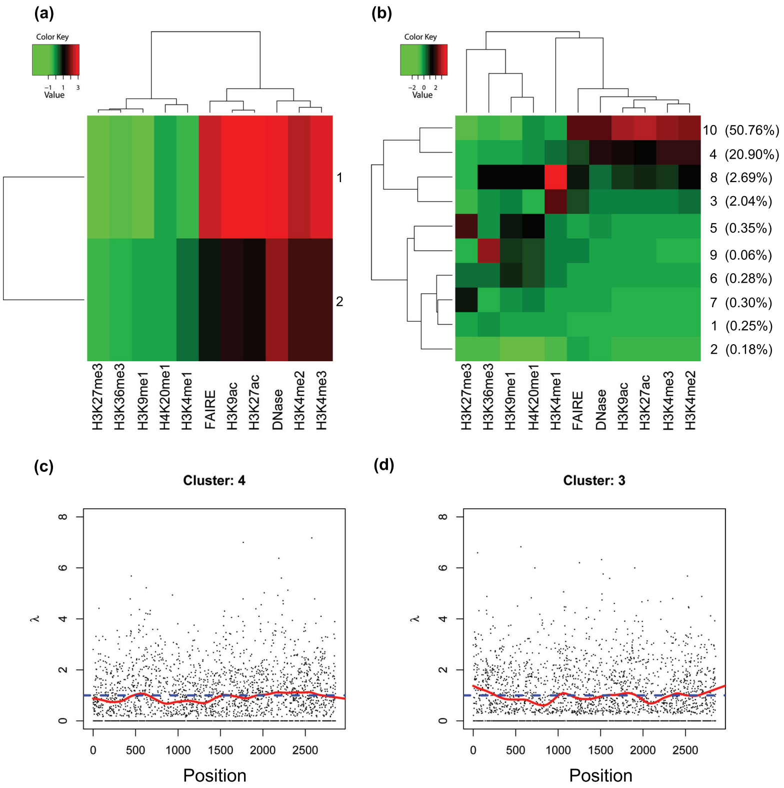

Fig. S5 K-means clustering of GABP motif sites based on chromatin surrogate signals $\mathbf{x}_s$. (a) Motif sites bound by GABP were determined using GABP ChIP-seq. The bound motif sites were clustered based on the Euclidean distance. The plot shows results for $k = 2$ clusters. For each cluster and each surrogate, the average signal across all motif sites is shown. We tried different cluster numbers, and obtained similar results for different k (data not shown). (b) All GABP motif sites (including bound and non-bound sites) were clustered into $k = 10$ clusters based on $\mathbf{x}_s$. For each cluster, the percentage of motif sites that are bound by GABP based on GABP ChIP-seq is shown in the brackets. Clusters 10 and 4 are enriched in true GABP binding sites, and both clusters show patterns similar to (a). This indicates that most bound motif sites share a similar chromatin pattern. (c),(d) We cut the whole genome into 1Mbp non-overlaping bins. For each bin j, let A_j be the total number of motif sites within the bin. For each cluster k in (b), let a_{jk} be the number of motif sites in bin j that belong to cluster k. The proportion of cluster k motif sites in bin j is $p_{jk} = a_{jk}/A_j$. Let N_k be the total number of cluster k motif sites in the whole genome and N be the total number of all motif sites in the genome. $P_k = N_k/N$ is the genome-wide proportion of cluster k motif sites. For each bin j and cluster k, we computed $\lambda_{jk} = p_{jk}/P_k$, the relative enrichment of cluster k motif site in bin j compared to the genome-wide proportion. λ_{jk} is plotted across the genome for two representative clusters: (c) cluster 4, and (d) cluster 3. Different chromosomes are concatenated together in the plots. Since each bin has only a few motif sites (typically 2-4 on average) for each cluster, the bin level λ_{jk} has large statistical variation, therefore we used smoothing spline to obtain more stable estimates of λ_{jk} (red curve). We see that the red curves fluctuate around 1 (blue line) across the genome, and the fluctuation is relatively mild. For each cluster, there is no strong regionalized distribution of motif sites. Similar analyses were performed for all other TFs and clusters, and similar results were obtained (data not shown).

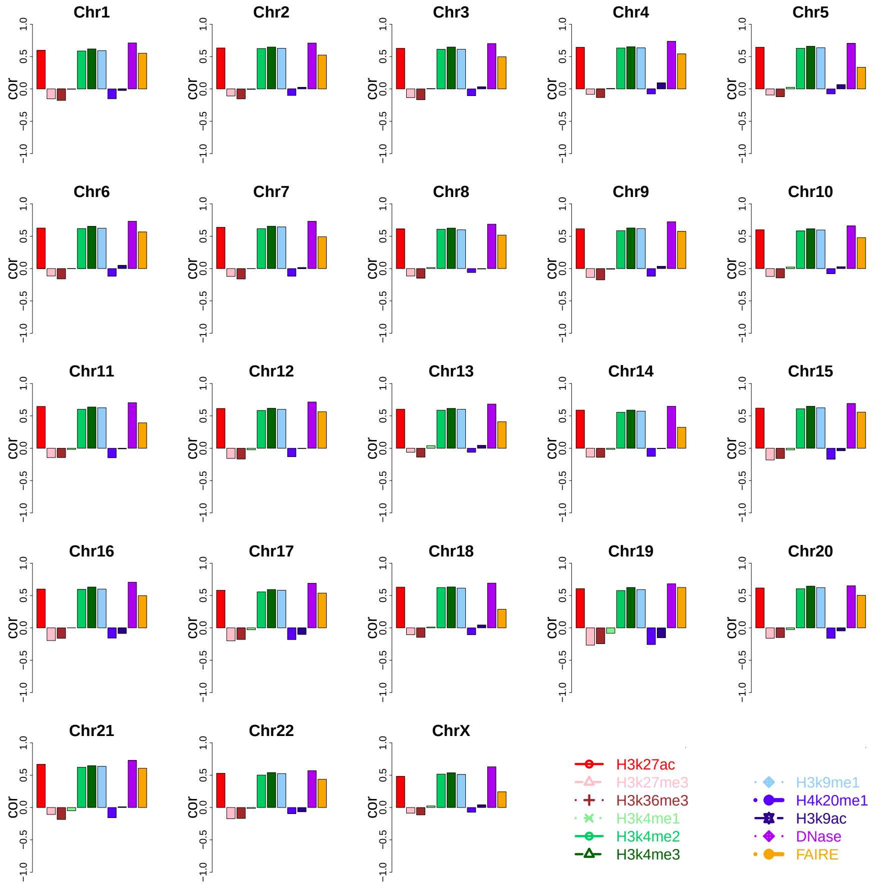

Fig. S6 Pearson correlation coefficients between GABP ChIP-seq binding intensity and various chromatin surrogates across all GABP motif sites in each chromosome. Different chromosomes show similar correlation patterns. GABP is a representative example. Similar analyses were performed for all other TFs and yielded similar results (data not shown).

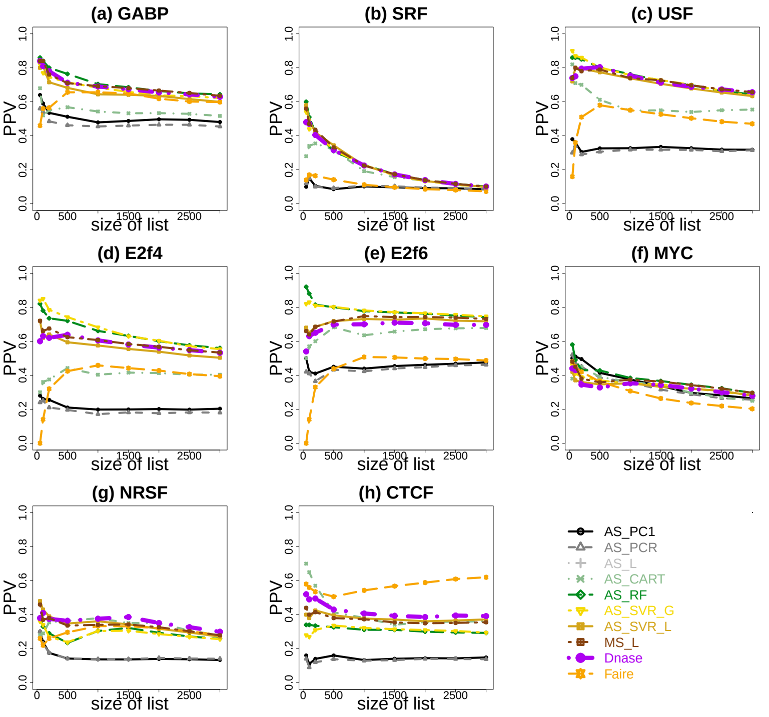

Fig. S7 Positive predictive value curves for predicting TFBSs in K562 based on models trained using EGR1. (a) Prediction for GABP; (b) prediction for SRF; (c) prediction for USF (d) prediction for E2F4; (e) prediction for E2F6; (f) prediction for MYC; (g) prediction for NRSF; (h) prediction for CTCF. Using other training and test TF pairs produced similar results (data not shown).

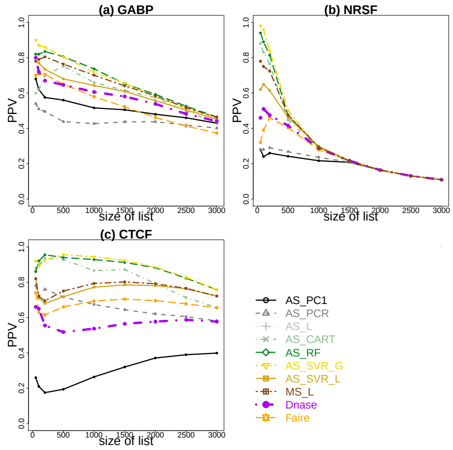

Fig. S8 Positive predictive values of prediction for chromosomes 17-22 and chromosome X by models trained using chromosomes 1-16 for (a) GABP; (b) NRSF; (c) CTCF. The training and test TFs are the same. Single surrogate predictions by DNase and FAIRE are also added for comparison. The three TFs shown are representative examples of all analyzed TFs.

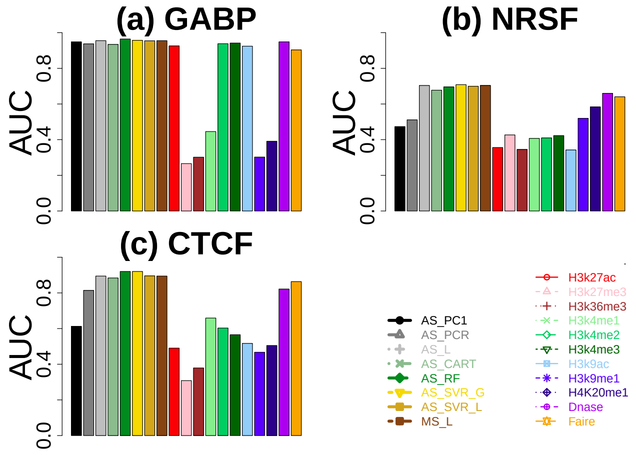

Fig. S9 AUC of prediction for chromosomes 17-22 and chromosome X by models trained using chromosomes 1-16 for (a) GABP; (b) NRSF; (c) CTCF. The training and test TFs are the same. Single surrogate predictions by DNase and FAIRE are also added for comparison. The three TFs shown are representative examples of all analyzed TFs.

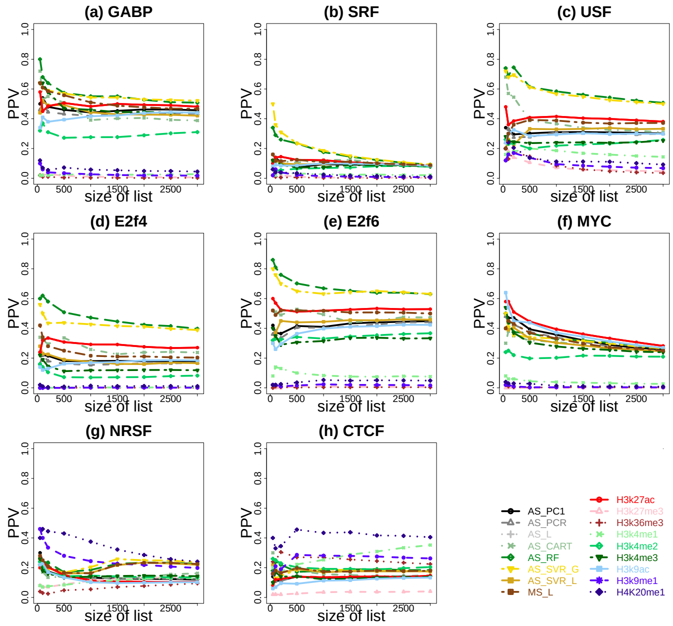

Fig. S10 Positive predictive value curves for predicting TFBSs in K562 based on models trained on EGR1 using only HM ChIP-seq data. (a) prediction for GABP; (b) prediction for SRF; (c) prediction for USF (d) prediction for E2F4; (e) prediction for E2F6; (f) prediction for MYC; (g) prediction for NRSF; (h) prediction for CTCF.

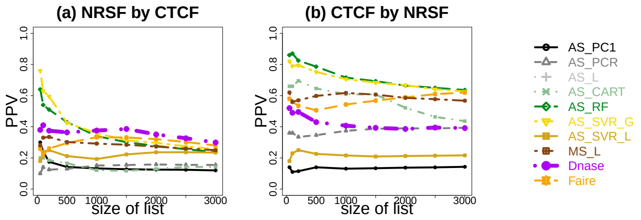

Fig. S11 Positive predictive value curves for (a) prediction for NRSF by models trained on CTCF using only HM ChIP-seqs and (b) prediction for CTCF by models trained on NRSF using only HM ChIP-seqs. Single surrogate predictions by DNase and FAIRE are also added for comparison.

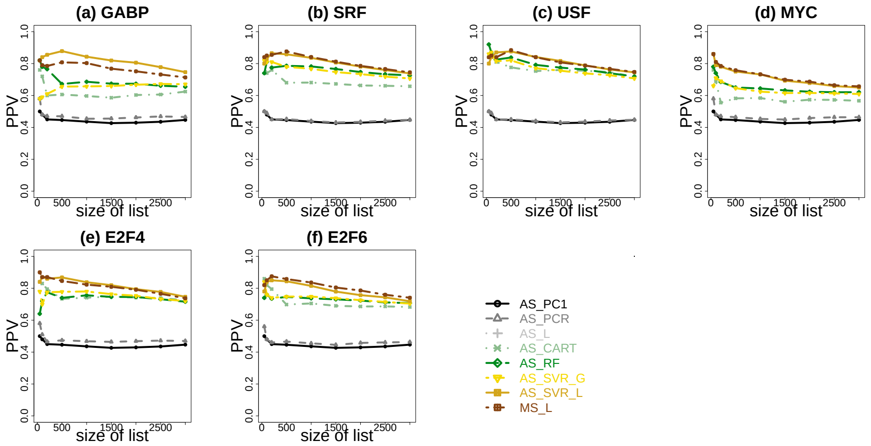

Fig. S12 Positive predictive value curves for prediction on EGR1 by models trained using ChIP-seq data from different labs. (a) Models trained using GABP (HudsonAlpha); (b) models trained using SRF (HudsonAlpha); (c) models trained using USF (HudsonAlpha); (d) models trained using MYC (UTA); (e) models trained using E2F4 (Yale); (f) models trained using E2F6 (Yale).
